# Electrochemical and power conversion performance of different counter electrode materials for flexible dye-sensitized solar cells

**DOI:** 10.1039/d3ra01974h

**Published:** 2023-07-06

**Authors:** Hina Pervaiz, Nadia Shahzad, Qasim Jamil, Muhammad Imran Shahzad

**Affiliations:** a U.S.-Pakistan Centre for Advanced Studies in Energy (USPCAS-E), National University of Sciences and Technology (NUST) H-12 Sector 44000 Islamabad Pakistan hpervaizphdf19.ces@student.nust.edu.pk; b Faculty of Chemistry and Chemical Technology, University of Ljubljana Vecna Pot 113 Sl-1000 Ljubljana Slovenia; c Nanosciences and Technology Department (NS&TD), National Centre for Physics (NCP) 44000 Islamabad Pakistan

## Abstract

Carbon dots and copper indium sulfide are promising photovoltaic materials, which have so far been fabricated mainly by chemical deposition methods. In this work, carbon dots (CDs) and copper indium sulfide (CIS) were separately combined with poly(3,4-ethylenedioxythiophene)-poly(styrenesulfonate) (PEDOT:PSS) for the preparation of stable dispersions. These prepared dispersions were used to produce CIS-PEDOT:PSS and CDs-PEDOT:PSS films using the ultrasonic spray deposition (USD) approach; furthermore, platinum (Pt) electrodes were fabricated and tested for flexible dye sensitized solar cells (FDSSCs). All the fabricated electrodes were utilized as counter electrodes for FDSSCs, and the power conversion efficiency of the FDSSCs reached 4.84% after 100 mW cm^−2^ AM1.5 white light was used to excite the cells. More investigation reveals that the improvement might be caused by the CDs film's porosity network and its strong connection to the substrate. These factors increase the number of sites available for the effective catalysis of redox couples in the electrolyte and facilitate the movement of charge in the FDSSC. It was also emphasized that the CIS film in the FDSSC device helps to generate a photo-current. In the beginning, this work shows how the USD approach can create CIS-PEDOT:PSS and CDs-PEDOT:PSS films and confirms that a CD based counter electrode film produced using the USD method is an interesting replacement for the Pt CE in FDSSC devices, while the results obtained from CIS-PEDOT:PSS are also comparable with standard Pt CE in FDSSCs.

## Abstract 2

Carbon dots and copper indium sulfide are promising photovoltaic materials, which have so far been fabricated mainly by chemical deposition methods.

## Graphical abstract

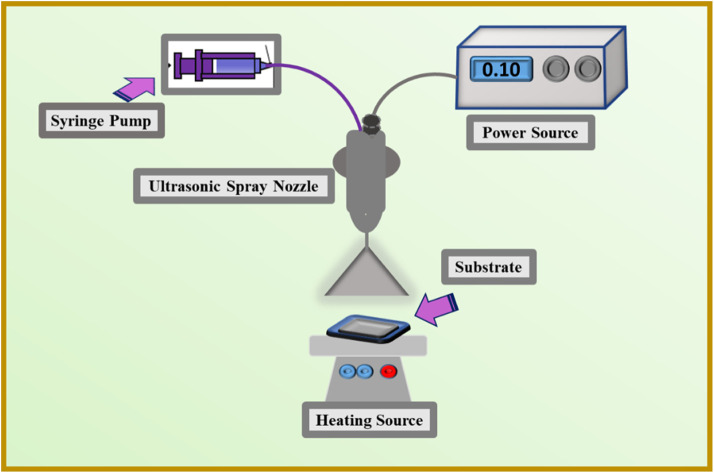

## 1. Introduction

Renewable energy technologies are suitable for combating the dilemmas of fuel depletion and global warming. Using photovoltaic technology to generate electricity is one method to mitigate reliance on fossil fuels. Photovoltaictechnologies seem to be the most renowned source of energy in terms of its effectiveness, efficiency, environmental friendliness, and affordability.^1,2^ In recent history, significant progress has been made in the field of solar energy. As of 2020, the price of generating PV energy has fallen below $0.05 per kW h, and that is equivalent to the cost of producing non-renewable resources. It is not inconceivable that, as a result of years of study and advancement of PV technology, the sun's energy would become incredibly affordable and fulfil human demands in the future. Third-generation PV devices are the most prevalent in terms of meeting current energy needs.^3,4^ Flexible dye sensitized solar cells (FDSSCs) of the third generation are low-cost and energy-efficient devices for addressing major global needs.^5,6^ As opposed to the pricey solar cells of the first generation, it is anticipated that FDSSCs will provide a low-cost alternative for meeting energy demands.^7,8^ FDSSCs also require a lower level of purity in the precursor material and are not overly sensitive to contaminants compared to other solar cell generations. However, the performance of this generation is inferior to that of the previous generation. Innovations are required to increase the efficiency of this new generation of devices.^9^

FDSSC consists of a dye-sensitized electrode (TiO_2_, ZnO, *etc.*), a redox-coupled electrolyte, and a counter electrode (CE).^10,11^ Among these components, CE has a highly important role in FDSSCs for efficient devices. High-performance DSSCs, on the other hand, usually require a noble metal, such as Pt, as the catalyst for the CE, which is cost prohibitive and so undermines one of the FDSSCs major focal features.^12^ Furthermore, it is doubtful if Pt will be capable of sustaining its long-term stability due to the possibility of a complex reaction between Pt and iodides when exposed to light. As a result, broadening the selection of CE materials is a difficult process. So far, several Pt replacements have been studied, including carbon-based compounds, conducting polymers, oxides, nitrides, and sulfides.^13–17^ Sulfides and carbon based materials have piqued the interest of researchers due to their versatility, such as electro-catalytic activity and excellent stability.^18^ Because of its high absorption coefficient, and consistent electro-catalytic activity, copper indium sulfide (CIS) and carbon dots (CDs) have been used as a light-harvesting component in thin-film solar cells and also as a CE material in FDSSCs.^19,20^

The quality of the film has a considerable impact on the performance of these devices. As a result, it is critical to investigate the film fabrication process. Spin coating, drip, and solution processing of films have been documented thus far because of their benefits of simplicity and compatibility with different kind of substrates.^21,22^ These techniques are capable of fabricating PVs with power conversion efficiencies that are equivalent to Pt-based FDSCCs using solution-prepared CIS films and carbon dots as the CE. However, further devices with improved efficiency than the Pt reference are hardly reported. This makes us more eager to investigate the production of CIS film utilizing the solution approach and the related FDSSC application.

The most straightforward and sophisticated technique for producing films through the solution process is ultrasonic spray deposition (USD). USD is a liquid atomization technique that primarily uses charge to disperse conductive liquid into small, uniform droplets.^23^ It also enables the creation of films with homogeneous morphology and a self-assembled microstructure. Modifying the flow rate or USD-time is also an efficient way to regulate the film's thickness.^24^ The hierarchical structure and porosity of the films produced by USD process may lead to better electrolyte percolation. A porous structure is preferred for CE because the catalytic mechanism for iodide regeneration favors more catalytic sites and electrolyte percolation channels.

Numerous studies have been conducted about the fabrication of FDSSC counter electrode films. Researchers have utilized a variety of ways to produce the CIS and CDs films, primarily for the photo-anode and a few for the cathode electrode and have tested the power conversion efficiency. In a previous study, we used electrophoretic deposition to prepare a CIS film for the photo-anode, and the DSSC efficiency achieved was 1.27%.^25^ Following the synthesis of nanoparticles and homogeneous ink, the CIS film was fabricated in this study using the ultrasonic spray method. In addition to testing the electrochemical and electrical performance of FDSSCs, the principal process parameters, including applied voltage, and deposition time, were also studied.

## 2. Experimental procedure

### 2.1 Materials

Glucose (C_6_H_12_O_6_), copper chloride dihydrate (CuCl_2_·2H_2_O), indium chloride tetra-hydrate (InCl_3_·4H_2_O), oleylamine (OAL), oleic acid (OA), sodium oleate, hexane, 2-methoxyethanol, ethanol, deionized water and an indium tin oxide and polyethylene naphthalate-based conducting plastic substrate (ITO-PET) were purchased from Sigma-Aldrich without further modification. Further sulfur (S) powder and acetylacetone were purchased from Merck. Further guanidinium thiocyanate (GuSCN), iodine (I_2_), 4-*tert*-butylpyridine, 1-methyl-3-propylimidazolium iodide (MPII), lithium perchlorate (LiClO_4_), lithium iodide (LiI) and acetonitrile for electrolyte preparation were purchased from Sigma-Aldrich. Polyvinylpyrrolidone (PVP), ethanol, titanium isopropoxide (TTIP) and hydrochloric acid (HCl) for working electrode were also purchased from Sigma-Aldrich. Ethylene glycol (C_2_H_6_O_2_), thiourea (CH_4_N_2_S), and acetylacetone were purchased from Merck.

### 2.2 Synthesis of carbon dots (CDs)

The synthesis was carried out using a microwave-based method to create the carbon dots solution. To initiate this procedure, glucose and deionized water were employed as a source of carbon and a solvent, respectively. After 30 min of stirring at room temperature, the process of manufacturing CDs got underway with the formation of an aqueous carbon solution that contained 8.9% carbon. After that, 10 ml of the solution was poured into a glass bottle and then microwaved for 9 min at medium power. The process of heating the solution in a microwave, which causes the solution to change color, ultimately results in the manufacture of CDs as the end product of the process (for example, from colorless to pale yellow). After that, the glass container was allowed to cool to room temperature for analysis and further use in the counter electrode fabrication.

### 2.3 Synthesis of CIS NPs

Copper chloride dihydrate, indium chloride tetra-hydrate, and sulfur powder were employed as precursors of Cu, In, and S, respectively. Sodium-oleate was used to make Cu-oleate and In-oleate independently. In addition, CIS nanoparticles were produced in a three-neck flask with 25 mL of oleylamine serving as the solvent. The flask also contained 0.5 mmol of Cu-oleate and 0.5 mmol of In-oleate. 1 mmol of sulfur powder was dissolved in 15 ml of oleylamine to make a separate sulfur solution, which was then swiftly introduced into the combination of metal oleate. After heating the flask at 80 °C for 30 min while maintaining a vacuum, the flask was then backfilled with argon after being linked to a Schleck line. The final solution became a dark brown color after being heated for 90 min at 180 °C. In order to precipitate the result, 10 ml of ethanol was added to the mixture after it had been cooled down to room temperature. The resulting mixture was subjected to several rounds of hexane washing before being centrifuged at 5000 rpm for 10 min. After some time, the dark precipitation began to develop. After that, the black precipitate went through a drying process at 100 °C, followed by an hour of heating at 300 °C.

### 2.4 Fabrication of counter electrodes

**Fig. 1:**
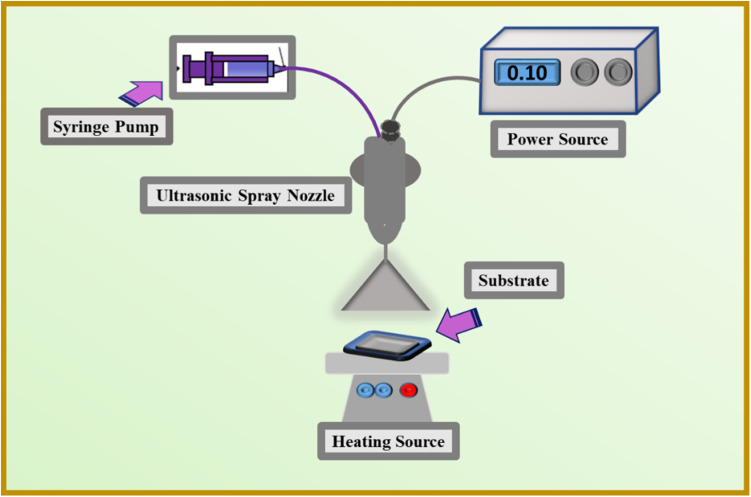
Schematic of counter electrodes fabrication *via* ultrasonic spray deposition (USD) process.

(I) A commercially available aqueous solution of PEDOT:PSS was used to prepare ink for the counter electrodes. First, we mixed the 30 vol% CDs solution in a 70 vol% solution of PEDOT:PSS and stirred for 60 min, forming a uniform and stable ink for the ultrasonic spray process. The substrate used for this purpose is ITO-PET plastic (2 × 2 cm^2^), which has been cleaned with isopropyl alcohol before deposition. For this process, the ink was sucked in 3 ml syringe, and this was placed on the syringe pump. Moreover, adjust the voltage using a high voltage power supply at the nozzle to atomize the (30 vol% CDs and PEDOT:PSS) ink, which was sprayed on a substrate with 100 μl min^−1^ deposition rate. Afterwards the deposited film was dried at ambient temperature and atmospheric conditions and then heated at 60 °C for 1 hour.

(II) The dispersion was made by combining 40 mg of synthesized CIS NPs with 2 ml of hexane and 26 mg of thiourea with 10 ml of ethanol, followed by 60 min of stirring. Then, the produced dispersion was combined with PEDOT : PSS (1 : 1 volume ratio), and the solution was stirred for two hours, resulting in a homogenous and stable ink for the ultrasonic spray procedure. Isopropyl alcohol was used to clean the ITO-PET substrate prior to deposition. For this process, the ink was sucked in 3 ml syringe, and this was placed on the syringe pump. Moreover, adjust the voltage using a high voltage power supply at the nozzle to atomize the CIS ink, which was sprayed on a substrate with 100 μl min^−1^ deposition rate. Later the deposited film was heated at 120 °C for 1 hour. The whole process for the fabrication of counter electrodes is shown in Fig. 1.

### 2.5 FDSSC's fabrication

For the working electrode of FDSSCs, a solution of (1 : 3) titanium isopropoxide in ethylene glycol methyl ether (C_3_H_8_O_2_) was applied on a conducting plastic ITO-PET substrate to achieve better connection between the substrate and the TiO_2_ coating. To produce a mesoporous, double-layered TiO_2_ film with a thickness of 15 μm, we use the well-known “doctor blade” approach. After that, the TiO_2_ film was treated at a temperature of 120 °C for 1 hour before being heated to 100 °C in a furnace at a rate of five degrees Celsius per minute. After heating and cooling the specimens to 80 °C, the TiO_2_/ITO-PET electrodes were placed in a N3 dye solution at a temperature of 55 °C for 1 hour. The prepared dye/TiO_2_/ITO-PET electrode was then separately paired with the CDs-PEDOT:PSS/ITO-PET, and the CIS-PEDOT:PSS/ITO-PET counter electrodes (prepared with the USD technique) and separated through a 25 μm thick PDMS membrane. As the electrolyte, we used 1.2 M DMPII, 0.35 M iodine, 0.1 M guanidinium thiocyanate, and 0.5 M 4-*tert*-butylpyridine in 10 ml of acetonitrile. This solution was then introduced into the gap between the working electrodes and the counter electrodes.

### 2.6 Characterization

To investigate the crystal structure and purity of the CIS nanoparticles and CDs, we utilized an X-ray diffractometer (XRD) that was equipped with CuKα radiation (*k* = 1.54051 Å) and a step size of 0.02°. A UV-Vis-NIR spectrophotometer was utilized to determine the optical characteristics, such as absorbance. A Fourier transform infrared (FTIR) spectroscopic experiment was done with an Agilent Cary 630 FTIR Spectrometer to find out information about the functional groups. Using a TESCAN MIRA3 field emission scanning electron microscope (FE-SEM), the surface topographies of the films were evaluated.

Using an electrochemical workstation (CHI660E), cyclic voltammetry (CV) was performed to evaluate the redox behavior of the electrode material in relation to tri-iodide reduction. This procedure was carried out utilizing a three-electrode assembly with a scan rate of 80 mV s^−1^ from −1.0 to 1.5 V, with produced films acting as the working electrode, Ag/AgCl as the reference electrode, and Pt wire as the counter electrodes. The electrolyte solution consists of 1 mM I_2_, 10 mM LiI, and 100 mM LiClO_4_ dissolved in acetonitrile. Using dummy cells with two identical electrodes and an electrolyte, the Tafel polarization curve and electrochemical impedance spectroscopy (EIS) were calculated. From −1.0 to 1.0 V, the Tafel plot was captured with a scan rate of 10 mV s^−1^. EIS was seen across the frequency range of 10^−1^ to 10^5^ Hz when a 5 mV amplitude AC signal was applied. Using a mask with a 0.25 cm^2^ window, the cell's active region was delineated.

The photovoltaic properties of nanostructure based FDSSCs were examined and analyzed. The open-circuit voltage and the short-circuit current were measured using a Newport Oriel that was connected with a Keithley PVIV 2400 source meter. These measurements were carried out under an irradiation of 100 mW cm^−2^. Following eqn (1) and (2), the output characteristics of the produced devices, namely the fill factor (FF) and the power conversion efficiency (*η*) of the solar cell, were calculated.1
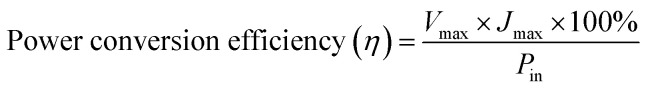
2
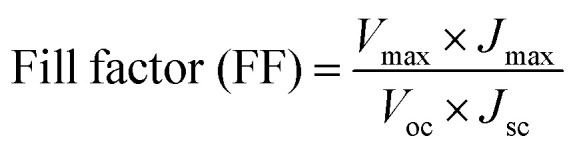


## 3. Results and discussion

### 3.1 Characterization of CIS NPs and CDs

**Fig. 2:**
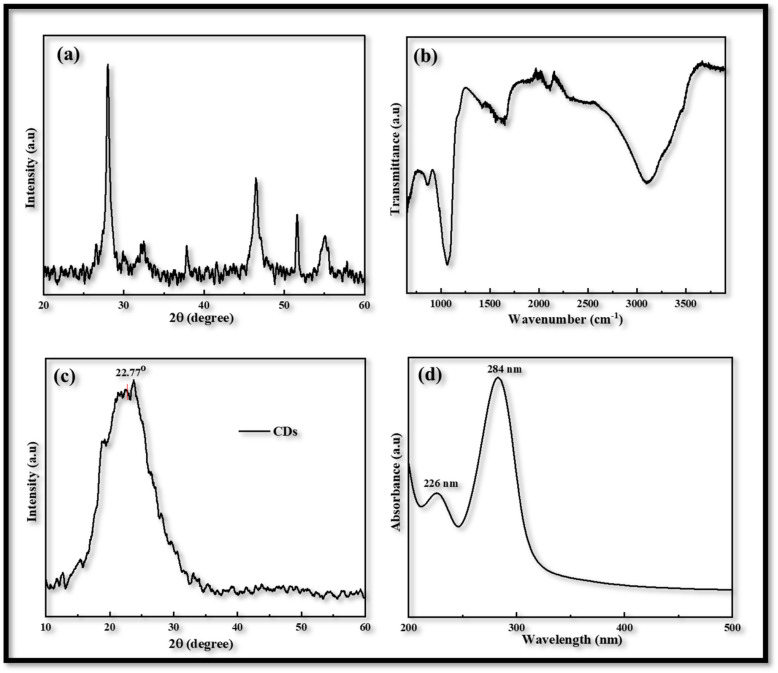
(a) & (c) X-rays diffraction of CIS nanoparticles and carbon dots (CDs) respectively, (b) FTIR spectrum of CIS nanoparticles, (d) absorbance spectrum of CDs in the range of 200 to 500 nm.

After the preparation of CIS NPs using Cu-oleate, In-oleate, and S powder in oleylamine solvent through a straightforward process as detailed in the Methodology section, the black powder that had been synthesized was analyzed for material characterization. The X-ray diffraction spectra of the CIS powder is illustrated in Fig. 2(a). The main three diffraction peaks located at 27.9°, 46.3°, and 54.9° 2*θ* values are well matched with the standard JCPDS no. 32-0339 pattern of tetragonal CIS. The presence of bi products is also observed in this spectrum. Further analysis was carried out using FTIR, and spectra of the CIS NPs is depicted in Fig. 2(b), revealing the presence of O–H bond at 3101 cm^−1^, stretching of carbonyl groups at 1630 cm^−1^, C=C bond at 1057 cm^−1^, C–O bond at 1057 cm^−1^, and further stretching at 859 cm^−1^ indicates the presence of indium oxygen bond and the formation of bi products as described in the XRD discussion.

CDs were investigated using XRD to learn about their structural properties. CDs XRD often reveal an amorphous character due to carbon atom disorderness. CDs XRD spectra of CDs normally exhibit a wide diffraction peak between 2*θ* values of 20° and 25° as shown in Fig. 2(c). The obtained products had an obvious diffraction peak at 22.77°, suggesting that carbonizing glucose would produce carbon structures. The peak is broad due to the small size of the CDs. The optical absorption spectra of CDs were studied to investigate their optical characteristics. The maximum absorption was observed in ultraviolet region of the electromagnetic spectrum. Fig. 2(d) illustrated the absorption of CDs at 226 nm and the maximum absorption at 284 nm, the absorption is correspond to Π–Π* transition of the C=C bond and n–Π* transition of the C=O bond respectively.^7^

### 3.2 Characterization of counter electrodes

Utilizing an electrochemical workstation, further cyclic voltammetry (CV) was carried out in order to evaluate the redox behavior of the produced electrodes that were obtained *via* the USD process. A three-electrode assembly with a scanning speed of 20 to 100 mV s^−1^ from −1.0 to 1.5 V was used for this procedure. The produced films were used as the working electrode, Ag/AgCl served as the reference electrode, and Pt wire was used as the counter electrodes. The electrolyte solution is comprised of 1 mM of iodine, 10 mM of lithium iodide, and 100 mM of lithium chloride in acetonitrile.

In voltammogram the peak-to-peak separation (*E*_pp_) and peak current density (*J*_c_) are two important parameters for comparing the electro-catalytic behavior of different counter electrodes. The peak-to-peak separation (*E*_pp_) is interrelated with reversibility of redox reaction and while the peak current density linked with reduction velocity. In the CV curves of both CDs-PEDOT:PSS/ITO-PET and CIS-PEDOT:PSS/ITO-PET counter electrodes, we obtained two pairs of redox peaks.

**Fig. 3:**
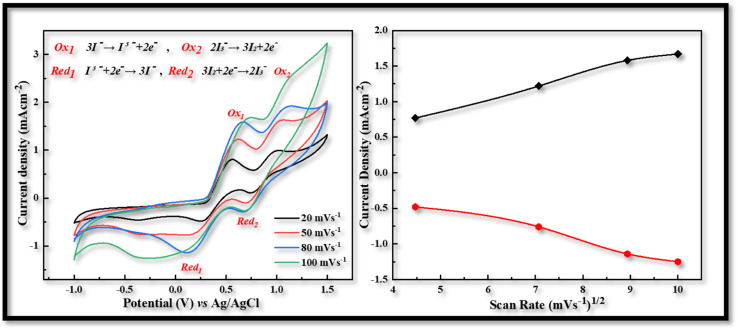
Cyclic voltammetry of CDs-PEDOT:PSS counter electrode with scan rate of 20 to 100 mV s^−1^ from −1.0 to 1.5 V.

The positive peaks are known as anodic peaks because they are relating to the oxidation of I^−^ in an electrolyte solution containing I_2_ (0.001 M), LiClO_4_ (0.1 M) and LiI (0.01 M), and the negative peaks are known as cathodic peaks because they are relating to the reduction of I_3_^−^ are given in Fig. 3.^26^

**Fig. 4:**
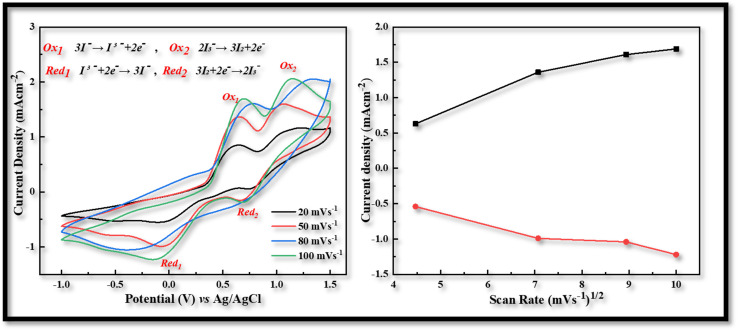
Cyclic voltammetry of CIS-PEDOT:PSS counter electrode with scan rate of 20 to 100 mV s^−1^ from −1.0 to 1.5 V.

According to the CV measurements, a high cathodic current density (*J*_c_) value indicates that the electrodes have a larger active surface area and conductivity, which is more advantageous for the reduction process of I_3_^−^ and improves the value of *J*_sc_ and the performance characteristics of FDSSCs.^27^ In contrast to other CV curves produced at 20 (−0.48 mA cm^−2^, 0.29 V), 50 (−0.78 mA cm^−2^, 0.52 V), and 100 (−1.25 mA cm^−2^, 0.97 V) mV s^−1^ scan rate, the CV curve obtained at 80 mV s^−1^ scan rate has a larger cathodic current density (−1.14 mA cm^−2^) and a smaller peak to peak potential difference (0.54 V) as shown in Fig. 3. So this kind of investigation showed the best behavior of CDs-PEDOT:PSS/ITO-PET counter electrode at 80 mV s^−1^ scan rate and also in the case of CIS-PEDOT:PSS/ITO-PET counter electrode as shown in Fig. 4, higher cathodic current density (−1.22 mA cm^−2^) was observed at 100 mV s^−1^ scan rate, but the lower peak to peak potential difference (0.71 V) was attained at 50 mV s^−1^ scan rate.

**Fig. 5:**
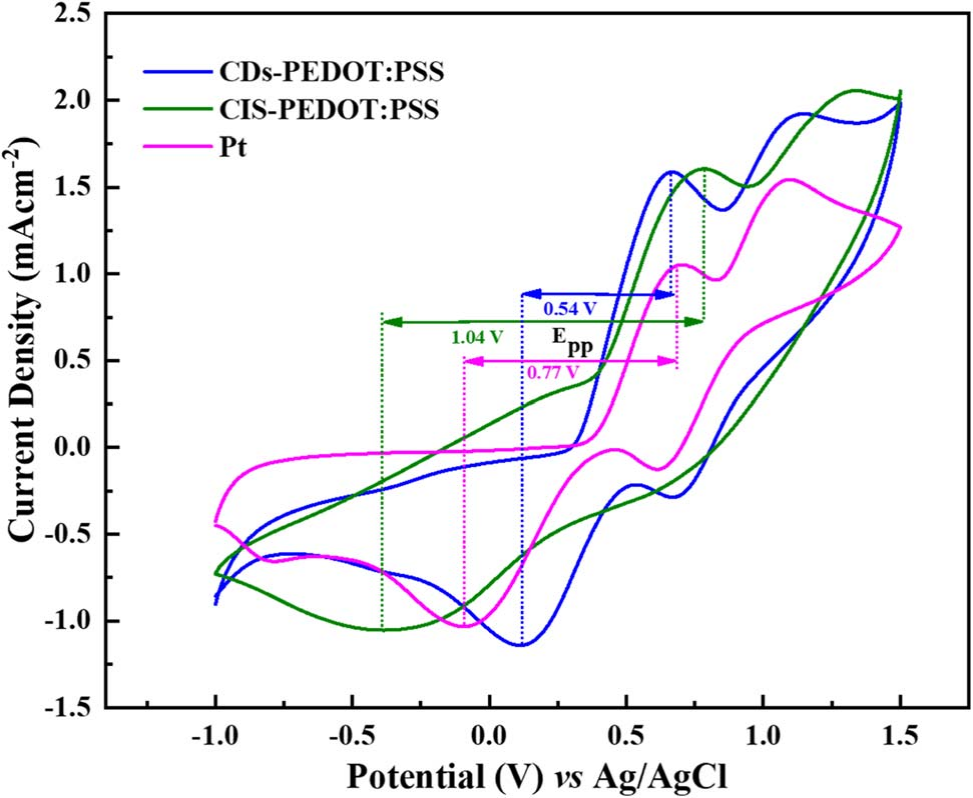
*Cyclic voltammetry curves o*f CDs-PEDOT:PSS, CIS-PEDOT:PSS, and Pt *counter electrodes at scan rate of* 80 mV s^−1^ from −1.0 to 1.5 V.

After the voltammogram measurements at different scan rate a comparison was made between the CDs-PEDOT:PSS/ITO-PET, the CIS-PEDOT:PSS/ITO-PET, and the Pt/ITO-PET counter electrodes at 80 mV s^−1^ scan rate. It was clearly noticed that in Fig. 5, the CDs-PEDOT:PSS/ITO-PET counter electrode showed higher cathodic current density(−1.14 mA cm^−2^) and lower *E*_pp_ (0.54 V) value at 80 mV s^−1^ scan rate instead of CIS-PEDOT:PSS/ITO-PET counter electrode and Pt/ITO-PET. As a result, the CDs-PEDOT:PSS/ITO-PET electrode has a larger active surface area and higher conductivity. This makes it more suitable for the reduction process of I_3_^−^ and results in an enhanced catalytic effect in contrast to the other two CEs. A comparison of *J*_c_ and *E*_pp_ values is given in Table 1.

**Table 1:** Comparison of *J*_c_ and E_PP_ values of different counter electrodes with literature work

Electrodes	*J*_c_ (mA cm^−2^)	*E*_pp_ (V)	References
CDs-PEDOT:PSS	−1.14	0.54	Current work
CIS-PEDOT:PSS	−1.04	1.04	Current work
Pt	−1.03	0.77	Current work
CuInS_2_/PEDOT:PSS	−1.23	0.80	23
PEDOT:PSS	−0.65	1.21	23
GD-10@hGO	−1.40	0.60	20
RGO	−0.66	0.52	12

**Fig. 6:**
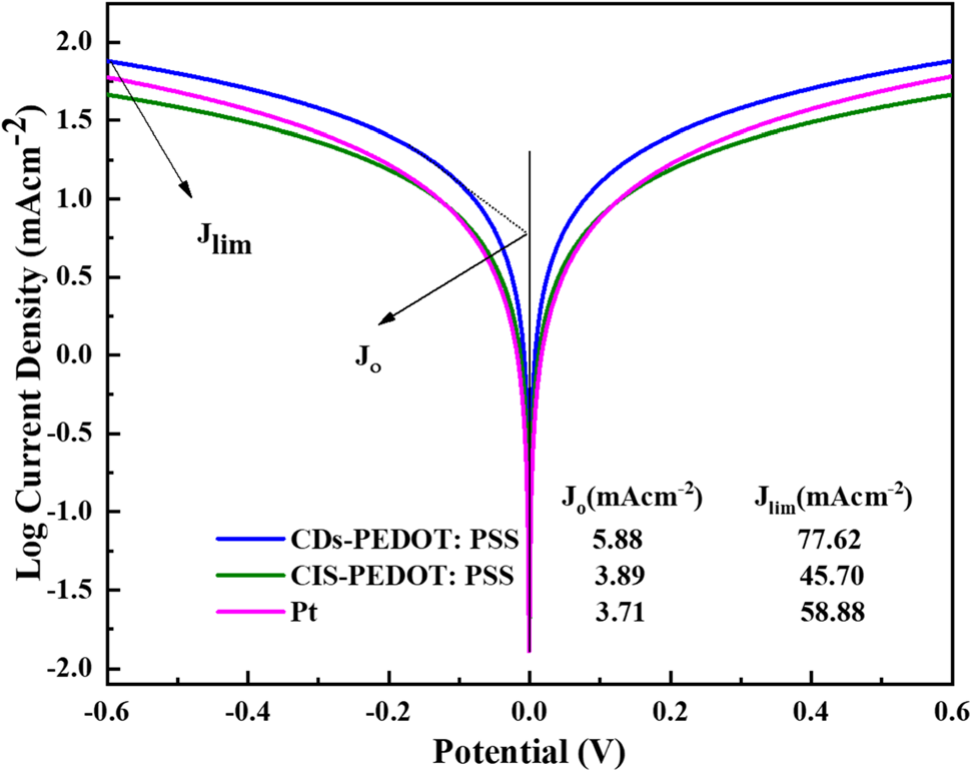
Tafel polarization plots of CDs-PEDOT:PSS, CIS-PEDOT:PSS, and Pt counter electrodes at from −0.6 to 0.6 V, and the values of *J*_o_ and *J*_lim_ is also given.

**Fig. 7:**
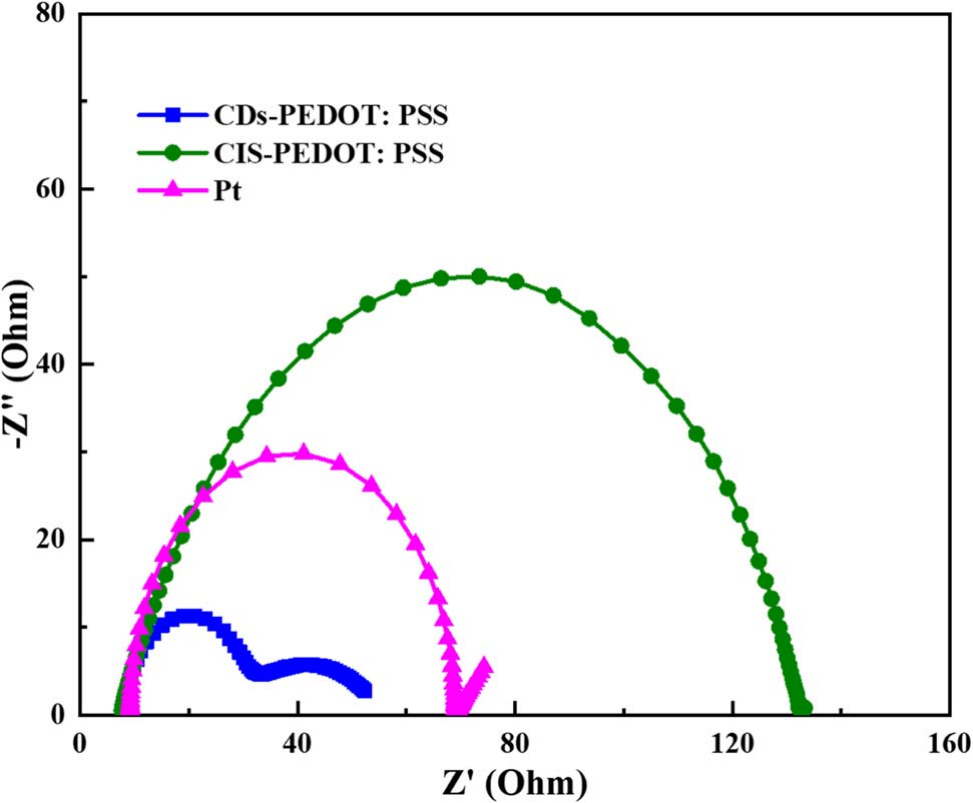
Nyquist plot of all the three counter electrodes (CDs-PEDOT:PSS, CIS-PEDOT:PSS, and Pt).

Further investigation was made through Tafel polarization and electrochemical impedance spectroscopy (EIS) on symmetric dummy cells of three different counter electrodes and a similar electrolyte that is used in the fabrication of solar cell devices. Fig. 6 shows the Tafel polarization curves of CDs-PEDOT:PSS/ITO-PET, the CIS-PEDOT:PSS/ITO-PET, and Pt/ITO-PET counter electrodes. In this mechanism the exchange current density (*J*_o_) and limiting diffusion current density (*J*_lim_) play crucial role because these parameters identify the electrochemical activity and diffusion ability of counter electrodes. From these curves, the exchange current densities (*J*_o_) were calculated from the interaction of cathodic branch with the equilibrium potential and the limiting diffusion current density (*J*_lim_) from the interaction cathodic branch with *Y*-axis. The exchange current density of CDs-PEDOT:PSS/ITO-PET are higher than the CIS-PEDOT:PSS/ITO-PET and the Pt/ITO-PET counter electrodes as shown in the Fig. 6. While the values of *J*_o_ and *J*_lim_ are comparable for other two CEs (Fig. 7).

According to Nyquist plot all the counter electrodes charge transfer resistance at electrolyte/counter electrode interface (*R*_ct_) values changes in the same way as their exchange current densities (*J*_oo_). In the literature there is a inverse relation between the *R*_ct_ and *J*_o_ described that higher the *J*_o_ values lower the charge transfer resistance at electrolyte/counter electrode interface. The CDs-PEDOT:PSS/ITO-PET counter electrode has higher *R*_ct_ rate as compared to other electrodes as shown in Table 2. In contrast, the limiting diffusion current density (*J*_lim_) has a positive relationship with the diffusion coefficient as reported in literature. Based on this, counter electrodes with a higher *J*_lim_ help to get a better diffusion speed. The CDs-PEDOT:PSS/ITO-PET counter electrode has the highest *J*_lim_ of all the counter electrodes, which means it can move I^−^/I_3_^−^ electrolyte more easily.

**Table 2:** Electrochemical and photovoltaic parameters of three different types of counter electrodes at ITO-PET substrates

Electrodes	*R*_s_ (Ω)	*R*_ct_ (Ω)			*C* (μF)	*Z*_W_ (Ω)	*J*_sc_ (mAcm^−2^)	*V*_oc_ (mV)	*η* (%)
		*R*_ct1_	*R*_ct2_						
CDs-PEDOT:PSS	8.05	20.45 27.19		0.98		0.0026	8.70	787	4.84
CIS-PEDOT:PSS	7.98	132		6.03		—	9.70	710	3.53
Pt	9.18	61.67		4.96		0.5167	10.7	790	4.41

**Fig. 8:**
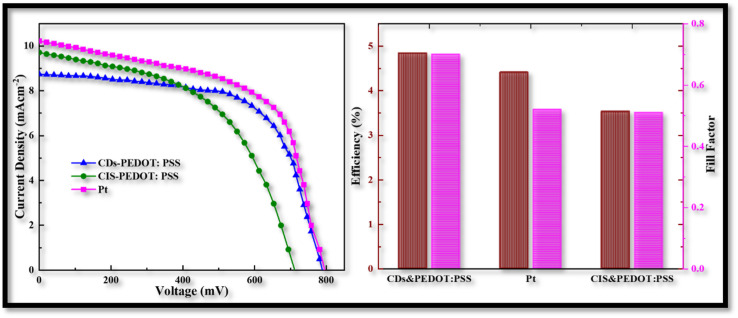
Power conversion efficiencies of CD-PEDOT:PSS, CIS-PEDOT:PSS, and Pt counter electrodes, and comparison of their fill factors.

Electrical properties were measured to acquire a better knowledge about the characteristics of the assembled CEs in FDSSCs. Fig. 8 illustrates the *J*–*V* characteristic curves of FDSSCs with CDs-PEDOT:PSS, CIS-PEDOT:PSS, and Pt counter electrodes. Fig. 8 summarizes the details of the photovoltaic parameters, such as fill factor (FF) and power conversion efficiencies (*η*), that were calculated using eqn (1) and (2).

The CDs-PEDOT:PSS CE based flexible cells shows superior PCE of 4.84% with the *V*_oc_ of 787 mV, *J*_sc_ of 8.7 mA cm^−2^, and FF of 0.70 which is higher than the Pt and CIS-PEDO:PSS based flexible cells. The improved PCE of the CDs-PEDOT:PSS based FDSSC is mainly due to the CDs structure which offers a larger surface area with a charge transport. These results are consistent with the electro catalytic measurements, the cell made with CDs-PEDOT:PSS CE have smaller *R*_ct_ and the *R*_total_ values compared to Pt and CIS-PEDOT:PSS CEs based FDSSCs. These results are also indicating that the CDs are helpful to improve the photoelectric performance which consistent with the Tafel data. And hence the performance of the CDs-PEDOT:PSS CE can be seen to be superior then other two CEs.

**Fig. 9:**
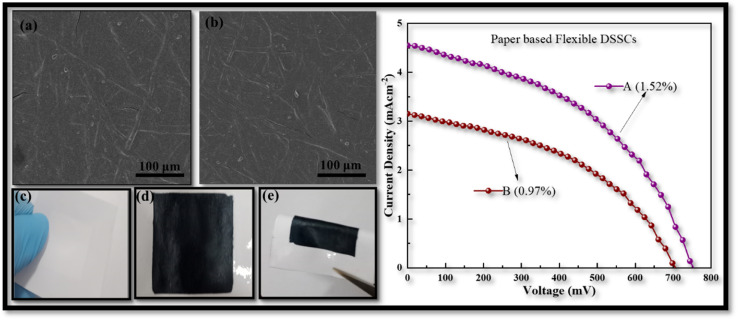
Illustration of paper based electrode (a) prepared from immersing the paper in the CDs-PEDOT:PSS ink, (b) prepared from USD process, (c) & (d) paper substrate without and with deposition, and (e) foldable. Also containing the PCE of paper based flexible electrodes.

To further investigate the benefits of the CDs in PEDOT:PSS, we also developed flexible paper-based counter electrodes for FDSSCs using TiO_2_/ITO-PET photo anodes. The paper based counter electrodes was prepared from two different techniques, (A) by ultrasonic spray deposition according to the above given method and (B) by immersing the paper substrate in CDs-PEDOT:PSS solution for 10 min and then dried at 60 °C for 1 hour. The counter electrodes comprised of flexible CDs-PEDOT:PSS/paper are shown in Fig. 9. SEM images of the paper electrodes prepared from above mentioned techniques are depicted in Fig. 9(a) and (b). The top view of these photos demonstrate that the paper substrate's porosity can be efficiently filled with the CDs-PEDOT:PSS composite. The surface of electrode A is smoother as compared to electrode B and the width of cracks is also smaller in the prior film, which results that the electrode A promotes effective electron transport inside the paper substrate's porous matrix.

While the photograph in Fig. 9(c)–(e) illustrate that this innovative material is compact, lightweight, and mold into various forms which is more useful in green energy production. The *J*–*V* curves of flexible paper-based FDSSCs are provided in Fig. 9. The electrical performance of the electrode prepared from USD technique is also better (1.52%) than the other one (0.97%). Compared to the cell made with the CIS-PEDOT:PSS/paper counter electrode (1.06%), the flexible DSSC made with ultrasonically CDs-PEDOT:PSS/paper counter electrode had a higher (1.52%) efficiency. The porous matrix of the paper substrate can be effectively filled with small sized carbon dots and polymer composite structures. This was attributed to improved charge transport in the less porous CDs and PEDOT:PSS paper electrodes. Although the device's efficiency is reduced when a paper-based substrate is used instead of an ITO-PET based substrate, this can significantly reduce the manufacturing costs of the FDSSC.

## 4. Conclusion

In summary, the CDs-PEDOT:PSS CE was successfully prepared on the ITO-PET and CP substrate *via* a ultrasonic spray deposition method and CDs structure is obtained *via* microwave assistant technique. These CDs-PEDOT:PSS offers potential prospect in the FDSSCs application which improved the redox ability for the reduction of I_3_^−^ to I^−^. Further, the use of CDs-PEDOT:PSS can enhance the diffusion rate of I^−^/I_3_^−^, reduce the charge transfer resistance and have better conduction of electricity than the CIS and Pt CEs, which provides a new choice for CE material. As a result, CDs-PEDOT:PSS/ITO-PET CE exhibits enhanced photoelectric performance with greater PCE of 4.84% in contrast to CIS-PEDOT:PSS/ITO-PET and Pt/ITO-PET CEs. All the results illustrate CDs-PEDOT:PSS can be an effective material to obtain advanced CE such that on CP with better electrical performance for FDSSCs. Hence, using a paper-based substrate as opposed to an ITO-PET-based substrate reduces the device's efficiency, but greatly reduces the FDSSC's production costs and plays a role in the production of biodegradable devices.

## Conflicts of interest

There are no conflicts to declare.

## Supplementary Material
